# Stimulator of Interferon Gene Agonists Induce an Innate Antiviral Response against Influenza Viruses

**DOI:** 10.3390/v16060855

**Published:** 2024-05-27

**Authors:** Hyun Jung Lee, Joo-Hoo Park, Il-Ho Park, Ok Sarah Shin

**Affiliations:** 1 BK21 Graduate Program, Department of Biomedical Sciences, College of Medicine, Korea University Guro Hospital, Seoul 08308, Republic of Korea; 2 Department of Otorhinolaryngology-Head and Neck Surgery, Korea University College of Medicine, Seoul 08308, Republic of Korea; 3 Upper Airway Chronic Inflammatory Diseases Laboratory, Korea University, Seoul 08308, Republic of Korea

**Keywords:** influenza virus, STING agonists, diABZI, macrophages, air–liquid interface cultures

## Abstract

The devastating effects of COVID-19 have highlighted the importance of prophylactic and therapeutic strategies to combat respiratory diseases. Stimulator of interferon gene (STING) is an essential component of the host defense mechanisms against respiratory viral infections. Although the role of the cGAS/STING signaling axis in the innate immune response to DNA viruses has been thoroughly characterized, mounting evidence shows that it also plays a key role in the prevention of RNA virus infections. In this study, we investigated the role of STING activation during Influenza virus (IFV) infection. In both mouse bone marrow-derived macrophages and monocytic cell line THP-1 differentiated with PMA, we found that dimeric amidobenzimidazole (diABZI), a STING agonist, had substantial anti-IFV activity against multiple strains of IFV, including A/H1N1, A/H3N2, B/Yamagata, and B/Victoria. On the other hand, a pharmacological antagonist of STING (H-151) or the loss of STING in human macrophages leads to enhanced viral replication but suppressed IFN expression. Furthermore, diABZI was antiviral against IFV in primary air–liquid interface cultures of nasal epithelial cells. Our data suggest that STING agonists may serve as promising therapeutic antiviral agents to combat IFV.

## 1. Introduction

RNA viruses continue to remain a threat to potential pandemics due to their rapid evolution. Potentiating host antiviral pathways to prevent or limit viral infections is a promising strategy to combat against pandemics. Influenza virus is a negative-stranded RNA virus known to be part of *Orthomyxoviridae* family, which causes a significant disease of the human respiratory system. The majority of yearly influenza virus epidemics, caused by the two currently circulating subtypes A(H1N1)pdm09 and A(H3N2), result in 5 million instances of severe disease and up to 650,000 deaths every year worldwide [1]. Given the medical importance of influenza-related disease burden, it is urgent to develop effect-ive antivirals needed for controlling influenza virus infection and disease.

Stimulator of interferon gene (STING), a critical innate immunity component located in the endoplasmic reticulum (ER), is essential for the activation of host innate immune responses against microbial infections [2]. STING is known to be activated upon binding of its cyclic dinucleotides (CDNs) ligand, which leads to the recruitment and activation of TANK-binding kinase 1 (TBK1) and other downstream factors for the induction of antiviral genes, such as IFNs and interferon-stimulated genes (ISGs). In addition, STING activation can induce autophagy-related gene 5 (ATG5)-dependent autophagy, which can restrict the replication of certain RNA viruses [3]. Furthermore, STING activation was found to effectively repress the replication of a broad range of DNA and RNA viruses [4]. Recently, a synthetic small molecule STING agonist, dimeric amidobenzimidazole (diABZI), was identified and elicited potent antiviral effects against multiple respiratory viruses including severe acute respiratory syndrome coronavirus 2, parainfluenza virus type 3, or human rhinovirus 16 [5,6,7]. The effect of STING agonists during influenza virus infection is yet to be determined.

In this study, we evaluated the antiviral effect of STING agonists against influenza viruses using human or mouse macrophages and primary air–liquid interface cultures of nasal epithelial cells. Our findings highlight a new functional role of STING agonists to reduce influenza replication and provide a new insight into the development of new antiviral agents to target respiratory viruses that continue to be an important source of human mortality and morbidity.

## 2. Materials and Methods

### 2.1. Cells and Viruses

THP-1 WT and STING knockout (KO) cells were obtained from Invivogen and A549 cells were obtained from the American Type Culture Collection. THP-1 cells were cultured in suspension using RPMI-1640 medium (Gibco) with 10% FBS and 1% penicillin-streptomycin and were differentiated with PMA (Sigma-Aldrich, St. Louis, MO, USA). To generate the murine bone marrow-derived macrophages (mBMDMs), 3–6-month-old C57BL/6 mice were euthanized, and bone marrow cells were collected by flushing the femurs and tibias with RPMI-1640 complete media containing 10% FBS, 1% penicillin/ streptomycin, 2 mM of L-glutamine, 0.05 mM of β-mercaptoethanol supplemented with 20% L929 supernatant containing M-CSF and L929 media preparation, as described [8]. Cells from each mouse were filtered through a 70 μm cell strainer and plated in untreated Petri dishes with 5 million cells per dish. Cells were maintained in a 37 °C incubator for seven days with media changes performed every two days.

A/H1N1 (Influenza A virus (H1N1) A/Korea/2008/H1N1), A/H3N2 (Influenza A virus (H3N2) A/Korea/2007/H3N2), B/Y (Influenza B virus Yamagata/Korea/2007) and B/V (Influenza B virus Victoria/Korea/2010) strains were obtained from Korea Bank for Pathogenic Viruses (Korea University School of Medicine, Korea), whereas A/PR8 (Influenza A virus (H1N1) A /Puerto Rico/8/1934/H1N1) was previously reported [9]. To measure infectious virus titer, a conventional plaque assay was conducted. In summary, MDCK cells were exposed to the virus in infection media (DMEM medium containing 7.5% bovine albumin fraction V, 1 mM HEPES, 2 μg/mL TPCK-trypsin and antibiotics) for one hour. Following the removal of the inoculum, the cells underwent three PBS washes before being covered with media containing 2% agar. After staining the cells with crystal violet at 72 h post-infection, the number of plaques was used to calculate the viral titers.

### 2.2. Antiviral Efficacy Assays

STING agonists (DMXAA, diABZI, 2′3′-cGAMP) were purchased from Invivogen. Cells were seeded in 96-well plates (1 × 10^5^ cells/well) and incubated overnight. To determine the antiviral effects of STING agonists, cells seeded in 96-well plates were incubated with varying dosages of STING agonists before or after IFV infection. After the removal of the mixture, a fresh serum-free medium was added. Trypan blue exclusion was used to track the vitality of the cells. In short, supernatants were extracted from infected and mock-infected (treated with medium alone) monolayers at different points following infection. MDCK cells seeded in 96-well plates were inoculated with diluted supernatants and incubated at 37 °C for 1 h. Serum-free media was then added to cells, which were incubated until the cytopathic effect was visible. At 72 h post-infection (hpi), cells were stained with Trypan blue solution (Invitrogen, Waltham, MA, USA). Viral titer was determined using the Spearman–Karber method and expressed as 50% tissue culture infectious dose (TCID_50_) units/mL. The antiviral effect of STING agonists was determined using TCID_50_ assay and the half-maximal effective concentration (EC50) values of diABZI were measured by GraphPad Prism 9.0.

### 2.3. Transfection

A549 were seeded in 6-well plates and allowed to grow until cultures were 70% confluent on the day of transfection. Transient transfections with STING-pUNO-HA plasmid (Invivogen, San Diego, CA, USA) were transfected by jetPRIME transfection reagent (Polyplus, Illkirch-Graffenstaden, France) [10] according to the manufacturer’s protocol. At 24 h post-transfection, cells were treated with bafilomycin A (BAF) or MG132 (MG) for 4 h. Cells were lysed, and lysates were subjected to immunoblotting to determine LC3 expression levels.

### 2.4. Cell Death Assay

Cells were stained with 5 μg/mL Propidium Iodide (PI) and 5 μg/mL Hoechst dye for 10 min. After double staining, cells were washed with DPBS. Stained cells were analyzed using the EVOS™ FL Auto 2 Imaging system (Thermo Fisher Scientific, Waltham, MA, USA). The % PI-positive cells were counted from at least 300 cells each condition compiled from at least three to six experiments and calculated by % PI-positive cells/total Hoechst positive cells.

### 2.5. Reverse Transcription-Quantitative PCR (RT-qPCR)

Total RNA was extracted using Direct-zol RNA mini Prep Kit (Zymo Research, Orange, CA, USA) and was reverse transcribed to generate cDNA using the Reverse Transcription system (Promega, Madison, WI, USA) for 1 h at 42 °C. RT-qPCR assays are performed using two-step approaches, cDNA synthesis followed by qPCR. Using a Power SYBR Green PCR Master Mix (Thermo Fisher Scientific), viral and host gene expression levels were measured. Primer sequences for host genes were previously reported [9,11] and viral genes are as follows. Polymerase A (PA)-F: CGGTCCAAATTCCTGCTGAT, (PA)-R: CATTGGGTTCCTTCCATCCA, Matrix protein 1 (M1)-F: AAGACCAATCCTGTCACCTCT GA, (M1)-R: CAAAGCGTCTACGCTGCAGTCC. The cycling parameters were as follows: 95 °C for 10 min, followed by 40 cycles of 30 s at 95 °C and 1 min at 60 °C. Glyceraldehyde 3-phosphate dehydrogenase (GAPDH) was used as a reference gene.

### 2.6. Immunoblot Analysis

Immunoblot analysis was performed as described previously [11]. Cells were harvested and lysed with RIPA buffer (Sigma) containing protease and phosphatase inhibitors (Roche, Basel, Switzerland). Proteins were separated by SDS-PAGE, transferred onto PVDF membranes, and blocked with 5% skim milk in TBS supplemented with 0.1% Tween-20 (TBS-Tw) for 1 h at room temperature. The membranes were then incubated with primary antibodies (Cell Signaling Technology, Danvers, MA, USA, 1:1000 dilution) at 4 °C overnight, followed by HRP-conjugated anti-rabbit or anti-mouse IgG secondary antibodies (Cell Signaling Technology) for 1 h at room temperature. Anti-β-actin (Abgent, San Diego, CA, USA) antibody was used as a loading control.

### 2.7. Enzyme-Linked Immunosorbent Assay (ELISA)

Supernatants from the basolateral side of the ALI culture were collected to measure the secretion level of cytokines. IL-6, IL-8, and IFN-β ELISA kits were purchased from R&D Systems. Assays were performed according to the manufacturer’s instructions. Absorbance at 450 nm was measured using a microplate spectrophotometer.

### 2.8. Characterization of Human Nasal Epithelial Cell (HNEC) Differentiation at Air-Liquid Interface (ALI)

Human nasal epithelial cells (HNECs) were collected from patients who underwent trans-sphenoidal pituitary tumor surgery. The study was approved by Korea University Medical Center Institutional Review Board and written informed consent was obtained from all patients in accordance with the Declaration of Helsinki. Cells were collected by scraping the mid-inferior turbinate with a brush and cultured in PneumaCult™-Ex Medium (Stemcell, Vancouver, BC, Canada). HNECs were amplified, washed with pre-warmed DPBS, and harvested using ACF Enzymatic Dissociation Solution (Stemcell). For the ALI culture system, the appropriate number of HNECs were seeded on an apical chamber of 0.4 μm transwell membrane inserts (Corning Mediatech, Manassas, VA, USA) with 0.5 mL PneumaCult™-Ex medium (Stemcell) and 1 mL PneumaCult™-Ex Medium was added to the basal chamber. The cells were incubated at 37 °C and media in both the basal and apical chambers were changed every other day for 2–4 days. When the cells reached 100% confluence, the medium was removed from both basal and apical chambers. 1 mL PneumaCult™-ALI Maintenance Medium (Stemcell) was added only to the basal chamber and changed every 2 days.

For apical infection, a fully differentiated primary ALI culture was inoculated with IFV, and STING agonists were added on the basolateral side. Cells of the ALI culture were incubated at 37 °C for 90 min followed by removal of the virus from the apical chamber. The infected cells of the ALI culture model were subject to further analysis to determine the antiviral effects of STING agonist treatment. To measure the effect of STING agonists on viral titer, supernatant from the basolateral side was titrated to determine viral titer on MDCK cells in the presence of 2 µg/mL TPCK-treated trypsin. Briefly, the virus was serially diluted 10 times and applied in quadruplicate to cell monolayers. Trans-epithelial electrical resistance (TEER) was monitored on a regular basis during the ALI differentiation of HNECs in order to characterize barrier function. After pre-warming DPBS to eliminate excess mucus, cells cultivated at the ALI were measured using an epithelial voltage/ohm meter (World Precision Instruments) and an STX-2 chopstick electrode.

### 2.9. Statistical Analysis

Statistical comparisons between the different treatments were performed using an unpaired two-tailed student’s *t*-test or Mann–Whitney test (Graphpad Prism, Boston, MA, USA), and *p* < 0.05 was considered statistically significant.

## 3. Results

### 3.1. STING Agonist diABZI Exhibited Potent Anti-IFV Activity in Mouse Bone Marrow-Derived Macrophages

**Figure 1:**
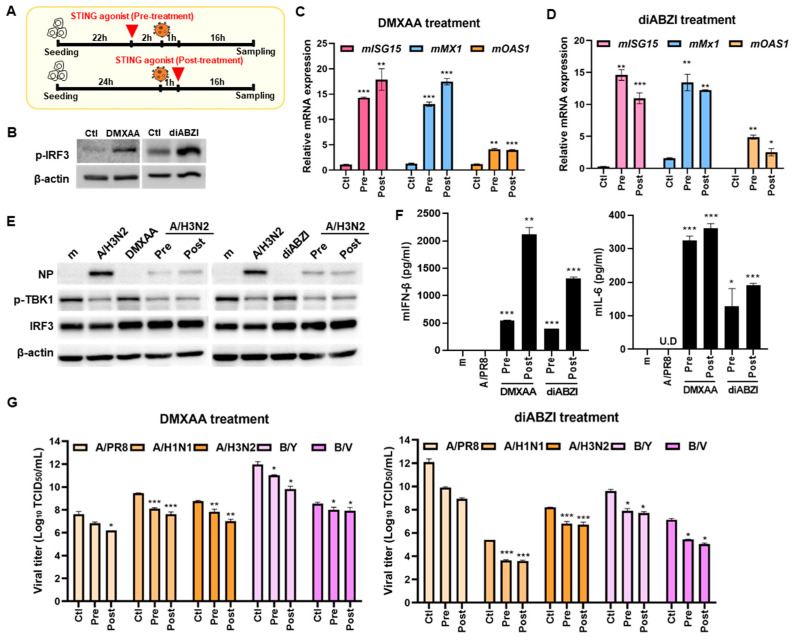
STING agonists are antiviral against IFV in mouse bone marrow-derived macrophages (BMDMs) (**A**) A schematic view showing that STING agonists were added to BMDMs before or after A/PR8 (MOI 5) or A/H3N2 (MOI 10) (**B**) BMDMs were exposed to DMSO control (Ctl), 25 μg/mL DMXAA or 3 μM diABZI for 4 h. Phosphorylation of IRF3 was measured by immunoblot analysis. Actin was used as the loading control. The blot shown is representative of three independent experiments. (**C**,**D**) BMDMs were treated with DMXAA or diABZI before A/PR8 (MOI 5) infection (Pre) or after A/PR8 infection (Post). RT-qPCR analysis of *Interferon-stimulated gene 15 (ISG15), MX Dynamin Like GTPase 1 (Mx1)*, and *O2′-5′-Oligoadenylate Synthetase 1 (OAS1)* mRNA were shown. Data are expressed the means ± SD of at least three independent experiments. * *p* < 0.05; ** *p* < 0.01; *** *p* < 0.001, versus control (Ctl; DMSO-treated) cells. (**E**) Protein levels of IFV NP, phospho-TBK1, and IRF3 were measured, and a representative blot is shown. (**F**) Secretion levels of mIFN-β and mIL-6 were measured by ELISA following DMXAA or diABZI pre- or post-treatment followed by A/PR8 infection. * *p* < 0.05; ** *p* < 0.01; *** *p* < 0.001, versus mock or A/PR8 infected cells. (**G**) Antiviral activities of STING agonists in BMDMs were determined by TCID_50_ assay. Data are expressed the means ± SD of at least three independent experiments. * *p* < 0.05; ** *p* < 0.01; *** *p* < 0.001, versus control (Ctl; DMSO-treated) cells.

The antiviral potential of STING agonist diABZI against SARS-CoV-2 and IFV in A549 cells has been previously investigated [12,13], however, the efficacy of STING agonists against IFV in macrophages is yet to be explored. We therefore investigated the potential anti-IFV activity of diABZI and DMXAA in mouse bone marrow-derived macrophages (BMDMs). First, STING agonists were added to mouse BMDMs before or after IFV A/PR8 infection (Figure 1A). The activity of STING agonists was confirmed by measuring the phosphorylation levels of interferon regulatory factor 3 (IRF3) (Figure 1B). Moreover, BMDMs were infected with IFV A/PR8 and mRNA levels of antiviral immunity-related genes were measured by RT-qPCR. Expression of the *Interferon-stimulated gene 15 (ISG15)*, *MX Dynamin Like GTPase 1 (Mx1)*, and *O2′-5′-Oligoadenylate Synthetase 1 (OAS1)* genes was significantly elevated by STING agonist treatment (Figure 1C,D). As expected, viral NP protein expression was greatly suppressed by diABZI treatment regardless of pre- or post-treatment (Figure 1E). On the other hand, STING agonist treatment led to elevated secretion levels of antiviral cytokine IFN-β, in response to IFV infection. Moreover, the induction of pro-inflammatory cytokine IL-6 was also upregulated upon STING agonist addition in BMDMs (Figure 1F). Next, we evaluated the antiviral activities of STING agonists by TCID_50_ assay. Figure 1G indicates that both DMXAA and diABZI show high antiviral efficacy against multiple strains of IFV, including A/PR8, A/H1N1, A/H3N2, B/Yamagata, and B/Victoria. Taken together, these findings indicate the possibility that STING agonist-triggered IFN signaling and activation contribute to controlling viral replication.

### 3.2. STING Agonist diABZI Exhibited Potent Anti-IFV Activity in THP-1 Cells

**Figure 2:**
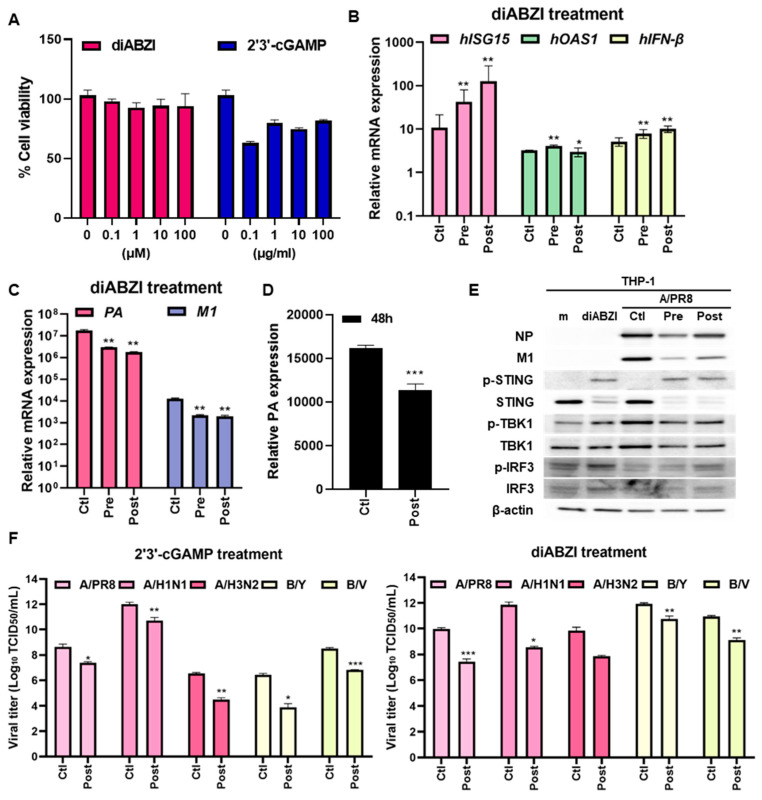
STING agonists inhibit IFV replication in human macrophages. (**A**) THP-1 cells were treated with various doses of STING agonist (diABZI, 2′3′-cGAMP) for 4h. % cell viability was measured. (**B**,**C**) THP-1 was treated with 3 M diABZI before A/PR8 (MOI 5) infection (Pre) or after A/PR8 infection (Post) for 16 h. Viral or host gene expression was quantitated, normalized, and analyzed by RT-qPCR (mean ± SD; *n* = 3). *Interferon-stimulated gene 15 (ISG15), O2′-5′-Oligoadenylate Synthetase 1 (OAS1)*, and *interferon-beta (IFN-β)* mRNA levels were shown, versus control (Ctl; non-treated) cells. (**D**) THP-1 was treated with 3 μM diABZI after A/PR8 (MOI 0.1) for 48 h. Viral gene expression was quantitated, normalized, and analyzed by RT-qPCR (mean ± SD; *n* = 3). (**E**) Protein levels of IFV NP, M1, phospho-STING/STING, p-TBK1/TBK1, and p-IRF3/IRF3 were measured, and a representative blot was shown. (**F**) Antiviral activities of STING agonists in THP-1 were determined by TCID_50_ assay. Data are expressed the means ± SD of at least three independent experiments. * *p* < 0.05; ** *p* < 0.01; *** *p* < 0.001, versus DMSO treatment (Ctl) cells.

Next, we determined the antiviral potential of STING agonist diABZI against IFV in THP-1 macrophages differentiated with PMA. THP-1 cells were incubated with various concentrations of STING agonists. A reduction in cell viability was observed in 2′3′ cGAMP-treated cells; however, the reduction was not dose-dependent and not statistically significant (Figure 2A). The activity of STING agonists was evaluated by measuring mRNA levels of antiviral immunity-related genes by RT-qPCR. The expression levels of *ISG15, OAS1*, and *IFN-β* were increased by STING agonist treatment regardless of pre- or post-treatment, whereas viral PA and M1 expression were downregulated by STING agonists (Figure 2B,C). We also performed a viral replication experiment with a lower MOI (MOI 0.1) and a long post-infection incubation period (48 h). The result still shows that IFV replication was significantly downregulated by diABZI post-treatment, similar to Figure 2C (Figure 2D). These findings were consistent with the results obtained with immunoblots in that viral NP and M1 expression were suppressed but phosphorylation levels of STING, TBK1 and IRF3 were elevated by diABZI (Figure 2E). As shown by Figure 2F, STING agonist treatment significantly attenuated the viral titer of multiple strains of IFV, including A/H1N1, A/H3N2, B/Yamagata, and B/Victoria. We also measured the half-maximal effective concentration (EC50) values of diABZI using Prism software. Compared to the 1.89 μM EC50 value of diABZI in THP-1, diABZI showed a similar potency, with an EC50 value of 1.34 μM in MDCK cells. Altogether, diABZI exhibits a potent antiviral efficacy against IFV in both human and mouse macrophages.

### 3.3. The STING Antagonist H-151 Exhibited an Opposite Effect in THP-1

**Figure 3:**
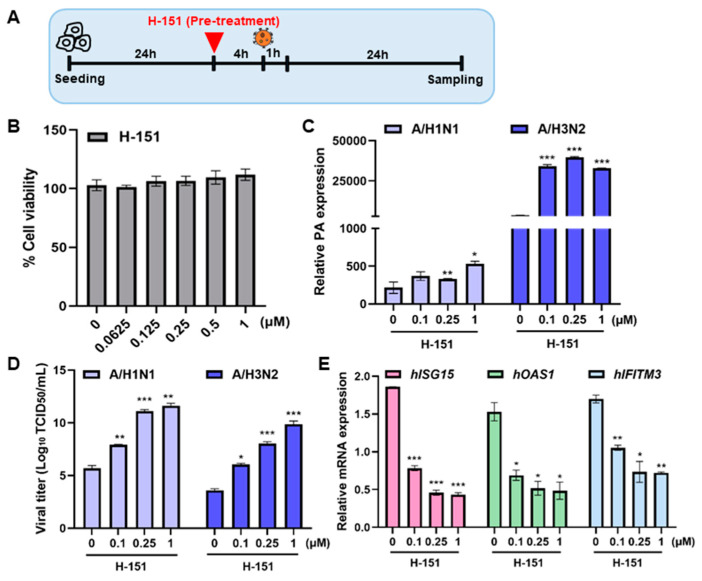
STING antagonist upregulates IFV replication. (**A**) THP-1 cells were treated with various doses of STING antagonist (H-151) for 4 h and infected with A/H1N1 or A/H3N2 (MOI 10). (**B**) Cell viability of various doses of H-151 treatment in THP-1 cells is shown. (**C**) THP-1 was exposed to 0, 0.1, 0.25, 1 μM of H-151 for 4 h and infected with A/H1N1 or A/H3N2. Viral PA expression level was quantitated, normalized, and analyzed by RT-qPCR (mean ± SD; *n* = 3). (**D**) Viral titer was measured by TCID_50_ assay (**E**) *Interferon-stimulated gene 15 (ISG15), O2′-5′-Oligoadenylate Synthetase 1 (OAS1)*, and *interferon-induced transmembrane protein 3 (IFITM3)* mRNA levels were quantitated, normalized, and analyzed by RT-qPCR (mean ± SD; *n* = 3). * *p* < 0.05; ** *p* < 0.01; *** *p* < 0.001, versus control (Ctl; non-treated) cells.

H-151 is a potent, selective and covalent antagonist of STING that has noteworthy inhibitory activity both in cells and in vivo [14,15]. The pharmacological STING antagonist H-151 was used to inhibit its activity by covalently binding to STING at the transmembrane cysteine residue 91, blocking STING palmitoylation. The effect of H-151 during IFV infection was tested using THP-1 cells (Figure 3A). First, various doses of H-151 were added to differentiated THP-1 cells and no cytotoxicity was observed at <1 μM (Figure 3B). Then, we added H-151 4 h before A/H1N1 or A/H3N2 infection. The effects on viral gene expression or viral titer were determined using RT-qPCR and TCID_50_ assay, respectively. Viral PA expression levels were significantly upregulated upon H-151 pre-treatment in IFV A/H1N1 or IFV A/H3N2 -infected cells (Figure 3C). Viral titers were also significantly enhanced upon H-151 treatment, as determined by TCID_50_ assays (Figure 3D). On the other hand, H-151 exerted an opposite effect on *ISG15*, *OAS1* and *IFITM3* gene expression, as shown in Figure 3E. In particular, H-151 dose-dependently attenuated ISG gene expressions, showing that STING activation is necessary for antiviral immunity against influenza viruses.

### 3.4. STING Restricts IFV Replication

**Figure 4:**
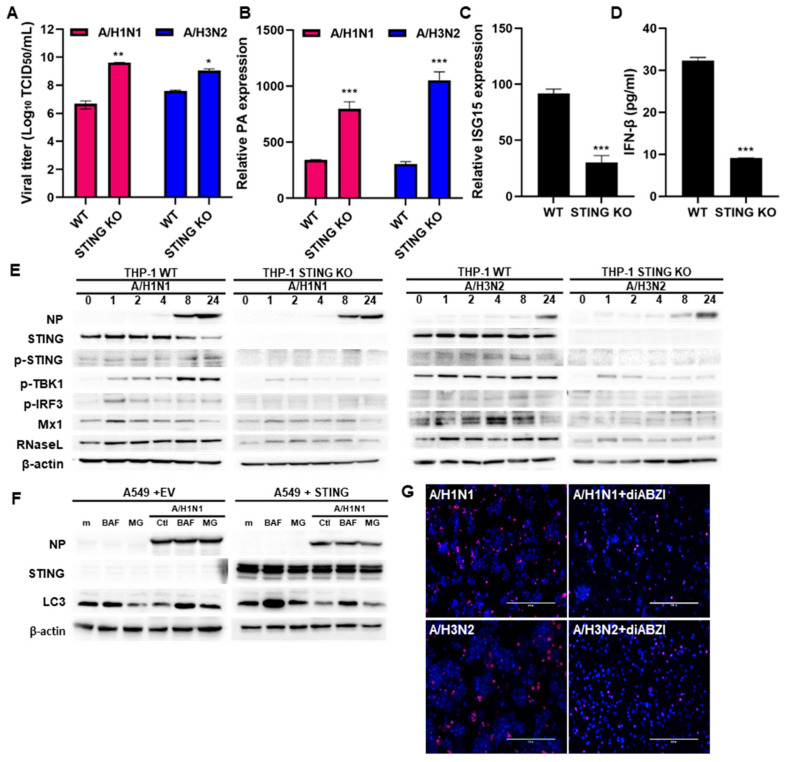
STING restricts IFV replication (**A**) Wild-type (WT), and STING knockout (KO) THP-1 cells differentiated with PMA for 2 days were infected with A/H1N1 or A/H3N2 (MOI 10). (**A**) Viral titer was measured by TCID_50_ assay. (**B**,**C**) RT-qPCR analysis of viral PA and host ISG15 mRNA was performed. Data are expressed as the means ± SD of at least three independent experiments. (**D**) Cell-free supernatant was tested for IFN-β by an ELISA method, versus THP-1 WT cells. (**E**) WT and STING KO THP-1 cells were infected with either A/H1N1 or A/H3N2 for indicated times. The protein levels of viral NP, phospho-STING/STING, phosho-TBK1, phospho-IRF3, Mx1 and RNaseL were analyzed by immunoblot analysis. The blot shown is representative of three independent experiments. (**F**) A549 cells were transfected with HA-STING plasmid for 24 h and then infected with A/H1N1 (MOI 10) for 24 h, and subsequently treated with 200 nM bafilomycin A (BAF) or 10 μM MG132 (MG) for 4 h. IFV NP and autophagy-related protein (LC3B) were detected by immunoblotting. (**G**) THP-1 cells were infected with A/H1N1 or A/H3N2 and treated with STING agonist (diABZI, 3 μM) for 16 h and PI/Hoechst staining was performed to measure cell death and examined using a double staining apoptosis assay (Hoechst33342/PI), * *p* < 0.05; ** *p* < 0.01; *** *p* < 0.001.

To further investigate the role of STING in controlling IFV replication, we analyzed IFV replication efficiency in THP-1 cells in which STING was deleted by CRISPR/Cas9 technology. Knockout efficiency was confirmed by immunoblot (Figure 4E). Subsequently, STING-silenced THP-1 cells were infected with IFV for 24 h at an MOI of 1. We found that the absence of STING remarkably facilitated IFV production (Figure 4A) and promoted viral RNA replication (Figure 4B). On the other hand, STING deficiency decreased *IFN-β* or *ISG15* production (Figure 4C,D). Similar results were observed by immunoblot analysis. In response to both A/H1N1 and A/H3N2, the absence of STING led to higher expression of viral NP (Figure 4E). Recent studies have shown that STING-mediated autophagy is important for antiviral responses [16]. Bafilomycin A (autophagy flux inhibitor) and MG132 (proteasome inhibitor) were used to investigate whether STING-mediated autophagy restricts IFV replication. Bafilomycin A treatment resulted in LC3II accumulation and increased autophagy flux, but failed to affect the expression levels of viral NP, suggesting that STING is less likely to control IFV replication via autophagy induction. Furthermore, MG132 failed to rescue STING-induced inhibition of IFV replication, and no change in the levels of LC3B-II was observed (Figure 4F). Lastly, we measured if STING agonists can prevent the induction of cell death caused by IFV infection in THP-1 cells. Figure 4G indicates that diABZI prevents IFV-induced cell death, suggesting that STING is essential for restricting IFV replication.

### 3.5. The Effect of STING Agonists in ALI Culture

**Figure 5:**
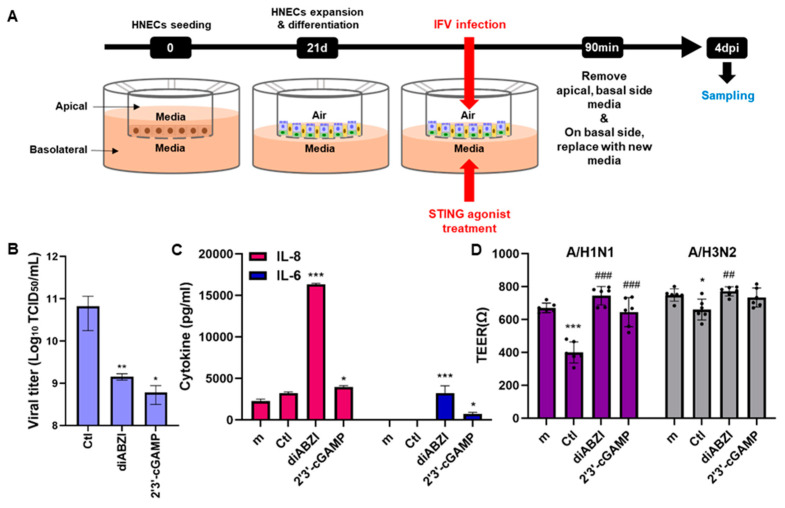
Antiviral effect of STING agonists was confirmed by the ALI culture model. (**A**) Schematic representation of the experimental design. HNECs of the ALI culture were treated with IFV (A/H1N1, MOI 10) at the apical side. STING agonist (3 μM diABZI, 1 μg/mL 2′3′-cGAMP)-containing media was also added in the basolateral side. After 90 min incubation, apical side media was removed, and basolateral side media was changed with fresh media containing STING agonists. After that, cells were incubated for 4 days and supernatants from basolateral side were subject to further analysis. (**B**) Virus titer was measured by TCID_50_ assay. (**C**) IL-6 and IL-8 cytokine secretion level was measured by ELISA. Mean + SD, *n* = 4, when compared to the control. (**D**) Trans-epithelial electrical resistance (TEER) plotted over time for HNECs of the ALI culture, Results in Ω are mean values of quintuplicate. Data was analyzed using a Mann–Whitney test. * *p* < 0.05, ** *p* < 0.01, and *** *p* < 0.001, versus m (mock infection only), ## < 0.01, ### < 0.001, versus Ctl (Virus infection only).

We previously showed that cells cultured at the HNECs of the ALI were susceptible to infections with IFV and HCoV, as shown by the viral replication levels and virus-induced loss of tissue integrity [9]. The therapeutic potential of STING agonists in inhibiting viral infection was further investigated using the ALI culture infection model. IFV was added to the apical side and STING agonists (diABZI or 2′3′-cGAMP) were treated in the basolateral side. The cell was incubated with the virus and STING agonist for 90 min and then the incubated media were removed and the fresh medium was added (Figure 5A). There was a significant suppression of viral titers when STING agonists were co-treated at the time of infection with IFV, in addition to an increase in cell viability (Figure 5B). On the other hand, STING agonists activated pro-inflammatory signaling pathways leading to increased cytokine production (Figure 5C). We also measured the barrier integrity of nasal tissues by trans-epithelial electrical resistance (TEER) over time as the ALI was being established. As expected, IFV infection led to a significant reduction in TEER, suggesting the disruption of tissue integrity, whereas the addition of diABZI or 2′3′-cGAMP contributed to the recovery of TEER values similar to the mock control (Figure 5D). These findings demonstrate that STING agonists have antiviral effects in IFV infection of the human nasal epithelium and highlight the potential of STING agonists to be developed as a safe and effective drug to prevent and treat infections with respiratory pathogens.

## 4. Discussion

Cyclic dinucleotide (CDN) and non-nucleotidyl STING agonists are being developed as therapeutic targets of cancer immunotherapy. Recent studies reveal the most potent, broad-spectrum antiviral function of STING agonists. Here, we describe that STING agonists inhibit the replication of multiple strains of IFV, including A/H1N1, A/H3N2, B/Yamagata, and B/Victoria.

2′,3′-Cyclic GMP-AMP dinucleotides (2′3′-cGAMP) acts as a key second messenger produced by cGAS by binding and activating STING [17]. Meanwhile, diABZI is a non-CDN of small-molecule amidobenzimidazoles identified to compete with 2′3′-cGAMP and activates the STING pathway [5]. Prior research demonstrated that diABZI has strong antiviral activities against coronaviruses, including SARS-CoV-2. It also has improved binding affinity and cellular functions in activating both human and murine STING [6,12,18]. In particular, diABZI treatment in the lung during SARS-CoV-2 infection triggers a quick, transient antiviral response via type I IFNs, NF-κB–driven cytokine production, and lymphocyte activation, resulting in inhibition of viral replication and prevention of severe respiratory disease [18]. Our findings indicate that diABZI exhibited antiviral activity with a potency comparable to that of 2′3′-cGAMP. When compared to other immunotherapies like recombinant IFN, using diABZI can offer greater advantages, including as lower cost, improved stability, room temperature storage, and the possibility of low-dose treatments being effective. Furthermore, a novel STING agonist, CDG^SF^ has been proposed by Wu et al. as a possible adjuvant for the SARS-CoV-2 vaccination [19]. Given that STING is essential for broad protection against multiple virus infections, it will be interesting to further evaluate the therapeutic antiviral potential of STING in vivo.

Recent research has revealed the dual function of STING, which controls IFN expression to limit DNA viruses and protein synthesis to inhibit RNA viral infection [20]. In this study, the antiviral activity of STING was found not to be mediated by autophagy, mitochondrial DNA or an inducible transcriptional response. However, STING regulates the translation of virus and host mRNAs. On the other hand, Wang et al. revealed that STING triggers autophagy by interacting with Rab7a and exerts anti-hantaviral effects instead of the type I IFN responses [21]. Typically, the cGAS-STING pathway reportedly plays a fundamental role in the production of IFNs and pro-inflammatory cytokines in response to DNA derived from invading microbial pathogens [22]. STING agonist treatment or STING overexpression significantly upregulated IFNs, pro-inflammatory cytokines (IL-1β), and interferon-stimulated genes (ISGs) (for example, Mx1 and ISG56) upon hantavirus infection. We examined the possibility of whether IFV replication is restricted by STING-mediated autophagy and found that STING is unlikely to govern IFV replication through the induction of autophagy. Considering that STING-mediated inhibition of IFV is coordinated by the production of type IFNs and proinflammatory cytokines via activation of the TBK1-IRF3 pathway, it is highly likely that diABZI’s mechanisms of action relied on IFN signaling.

As the first line of defense against microorganisms that are inhaled, the nasal airway epithelium is an important initial site for the interface between the environment and the host. We recently showed that the ALI culture system has proven to be a helpful platform for studying the interactions between respiratory pathogens and the host by in vitro reconstitution of a respiratory epithelium [9]. Infectivity titers quantified using TCID_50_ assay following infections with IFV demonstrate that cells used in the ALI culture model were successfully infected with the A/H1N1 strain. While diABZI and 2′3′-cGAMP both markedly reduced the ability of viruses to infect ALI cultures, they also enhanced the release of cytokines, which could potentially affect the barrier integrity within the ALI culture model and damage airway epithelium. As a result, it’s feasible that STING agonists will significantly reduce viral replication, but they’ll also need to be taken at a lower dosage to avoid having an excessively cytokine-producing effect. Thus, it will be important to further design an improvement of intracellular delivery of STING agonists and limit the potential of undesirable global inflammation.

Our data highlight the promising potential of STING agonists as a leading candidate for the therapeutic treatment of ongoing and emerging respiratory pathogens. Further investigation of the broad antiviral activity of STING agonists using the ALI culture system will be helpful in expanding the current regimen of FDA-approved antivirals against respiratory viruses of medical importance.

## Data Availability

The data presented in this study are available in the article and are available on request from the corresponding author.
